# Recent applications of artificial intelligence in cancer radiotherapy and immunotherapy: current status and future directions

**DOI:** 10.3389/fimmu.2026.1776472

**Published:** 2026-07-01

**Authors:** Qingmiao Shi, Mengjuan Xuan, Chi Lv, Zhibo Zhang, Xiaonan Geng, Di Huang, Xinjun Hu

**Affiliations:** 1 Department of Infectious Diseases, The First Affiliated Hospital, College of Clinical Medicine, Henan University of Science and Technology, Luoyang, China; 2 Henan Medical Key Laboratory of Gastrointestinal Microecology and Hepatology, Luoyang, China; 3 Department of Infectious Diseases, The First Affiliated Hospital of Zhengzhou University, Zhengzhou, China; 4 Department of Child Health Care, The Third Affiliated Hospital of Zhengzhou University, Zhengzhou, China

**Keywords:** artificial intelligence, cancer, drug discovery, immunotherapy, radiotherapy

## Abstract

Artificial intelligence (AI) enhances the precision, personalization, and efficiency of cancer treatment through deep learning and machine learning techniques. This review comprehensively examines the evolution of AI and its expanding applications in cancer radiotherapy, immunotherapy, and drug discovery and repurposing. In radiotherapy, AI enables automated medical image segmentation, thereby facilitating the accurate delineation of tumor targets. Furthermore, AI-driven feedback systems support clinicians in developing individualized treatment plans by offering real-time assessment of treatment safety and potential efficacy. In the context of cancer immunotherapy, AI integrates multi-omics data to advance the discovery of novel biomarkers, analyze the tumor immune microenvironment, and accurately predict responses to immune checkpoint inhibitors. Moreover, AI accelerates drug discovery and repurposing through virtual screening, protein structure prediction, and the identification of novel therapeutic targets. However, the true clinical value of these AI models depends heavily on their generalizability across diverse patient cohorts and their performance compared to standard clinical baselines. Despite these promising prospects, AI still faces challenges in clinical applications, such as insufficient data standardization, poor model interpretability, and a lack of ethical oversight, delaying its formal inclusion into standardized clinical guidelines. With the rapid growth of data volume and computational power, AI is expected to play an increasingly central role in cancer management, holding immense promise for improving patient outcomes and advancing precision oncology.

## 1 Introduction

Artificial intelligence (AI) is a broad field that integrates computer science, engineering and other disciplines (1). The primary objective of AI is to enhance machines’ ability for autonomous decision-making, continuous learning and the performance of complex tasks by simulating and extending human intelligence (2, 3). It consists of multiple subdomains. For instance, machine learning (ML), as a significant subfield of AI, focuses on the automated identification of patterns and regularities from large-scale datasets, thereby making predictions or decisions on unknown data without explicit programming (4). Deep learning (DL) is an important branch of ML that relies on multi-layer deep neural networks—such as Convolutional Neural Networks (CNNs) for spatial medical image analysis and Transformer architectures for complex sequential data—to achieve high-precision automatic feature extraction (5, 6). Over the past few decades, AI has exhibited rapid development, with significant advancements contributing to the progress in cancer management (7). Taken together, there is an urgent need to explore the application of AI in the filtration of substantial data for cancer diagnosis and treatment, especially in the context of the explosive growth of modern medical data (8).

Radiotherapy is widely regarded as one of the three fundamental strategies of cancer therapies, playing an important role in the management of both radical and palliative care for patients with malignancies (9). The lengthy duration and rigorous demands of radiotherapy workflow are key factors driving the integration of AI into this process (10). Notably, AI has enhanced the accuracy, efficiency and personalization of radiotherapy by the application in several key processes such as image analysis, treatment planning and automated procedures (11–13). Specifically, AI enhances the radiotherapy workflow by employing DL algorithms to automatically segment the high-risk areas of tumors and surrounding organs in medical images. This not only saves time but also improves accuracy (14). In terms of treatment plans, AI is driving the development of precision medicine, reducing the complexity of the plans and promoting the personalization of cancer treatment (15). For instance, AI demonstrates its potential in radiotherapy plans for specific cancer types, such as nasopharyngeal cancer, as shown in a recent report on an AI-empowered multistep radiotherapy workflow that has proven to be clinically practical (16).

Immunotherapy has emerged as a powerful clinical strategy for the treatment of cancer, which controls and eliminates cancers by reactivating the body’s anti-cancer immune responses (17, 18). Despite its effectiveness and promise, immunotherapy faces several unresolved challenges, such as the need to extend treatment efficacy to a broader spectrum of cancer types and accurately predicting responders (19, 20). The remarkable clinical benefits of immune checkpoint inhibitors (ICIs) have led to the need for accurately identifying patients likely to respond to treatment. The integration of AI into immunotherapy offers a promising approach for enhancing the efficacy and prediction of immunotherapy (21, 22). It is demonstrated that AI has played a significant role in the rapid development of immunotherapy through the deep integration of large-scale genomic, clinical, and imaging data (23). The integration of AI with multi-omics, including radiomics, pathology, and proteomics, enables the identification of potential therapeutic targets. This approach facilitates the advancement of personalized immunotherapy strategies and supports the optimization of individualized treatment plans (24).

Overall, AI demonstrates significant potential to transform the fields of immunotherapy and radiotherapy. By effectively integrating large-scale data with clinical workflows, AI is advancing the development of tailored cancer treatment strategies. Despite these promising strides, several challenges remain, including limited data accessibility, inadequate model interpretability, difficulties in clinical decision support, and the absence of well-defined regulatory and ethical guidelines. This review summarizes the latest advancements in the application of AI in cancer management, aiming to promote individualized treatment for cancer. Crucially, rather than merely describing AI applications, this review critically evaluates model performance, compares different AI methodologies, and discusses the translational gap between in silico success and real-world clinical value.

## 2 The historical evolution of AI development

**Figure 1:**
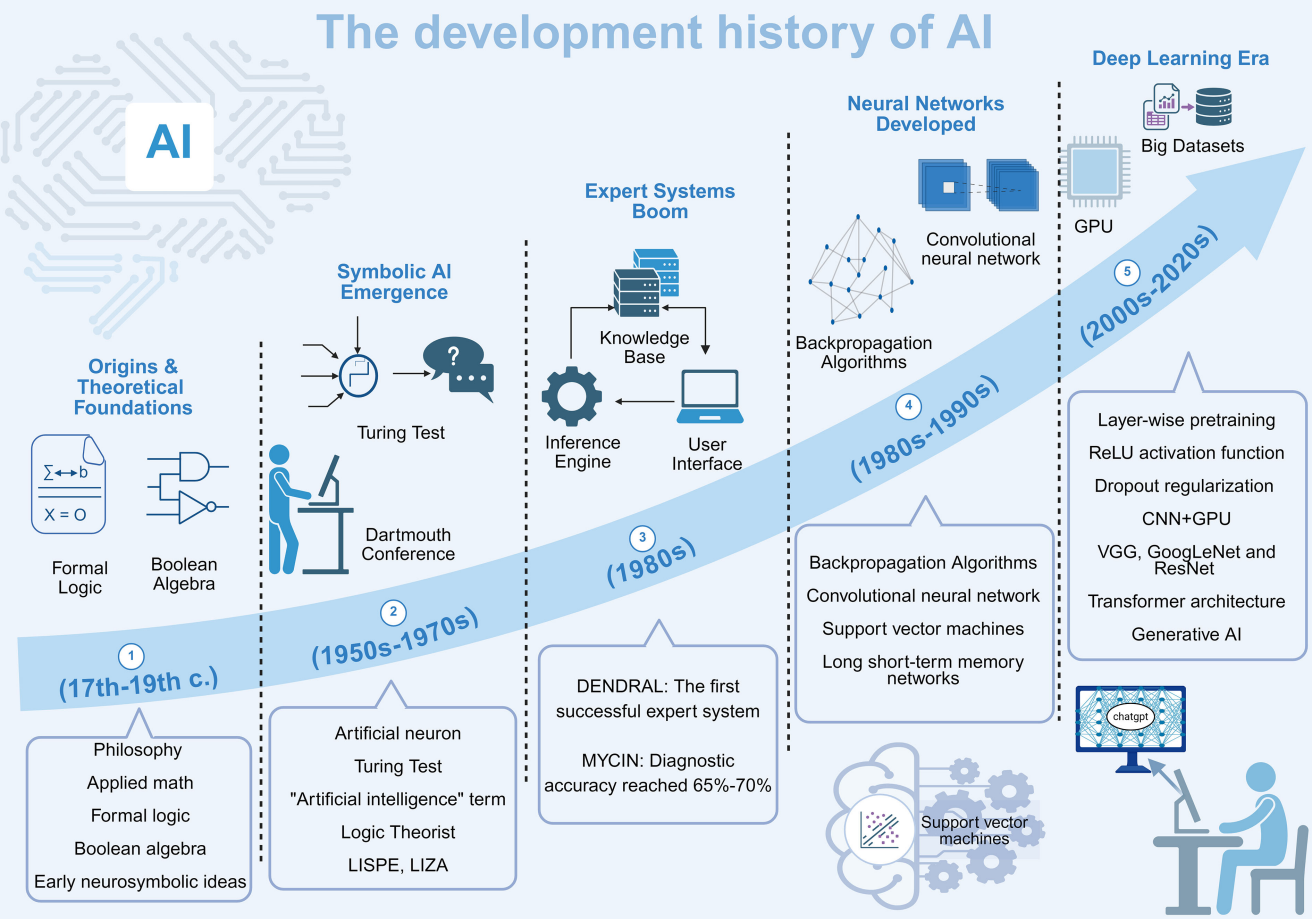
The historical evolution of artificial intelligence (AI), spanning from its theoretical foundations to expert systems, and finally into contemporary integrated AI frameworks.

Over the past few decades, AI has evolved from theoretical concepts and basic expert systems to sophisticated DL and large language model (**Figure 1**). In oncology, this evolution is important less as a chronology than as a methodological transition: modern AI can extract high-dimensional features from imaging, pathology, genomics, and clinical records, enabling applications in radiotherapy planning, immunotherapy response prediction, and drug discovery.

### 2.1 Origins and theoretical foundations (17th-19th centuries)

The conceptual origins of AI can be traced back to the 17th through 19th centuries. During this period, the symbolic operations established by Boolean algebra laid the early theoretical framework and conceptual foundation for modern computational logic and model reasoning capabilities (25, 26).

### 2.2 Emergence and development of symbolic AI (1950s-1970s)

In the middle of the 20th century, artificial neuron model and the Turing test marked the beginning of the AI era (27). Following the Dartmouth Conference, symbolic AI made significant progress in logical reasoning and programming languages, and the first AI programs emerged, providing a technical prototype for subsequent complex medical decision-making models (28, 29).

### 2.3 The rise and commercial application of expert systems (1980s)

In the 1980s, AI shifted toward domain-specific expert systems and began to explore applications in fields such as basic medicine. Notably, MYCIN achieved a diagnostic accuracy rate comparable to that of human physicians, validating the feasibility of AI in handling clinical knowledge bases and complex diagnostic logic (30, 31).

### 2.4 The development and limitation of neural network (1980s-1990s)

In the 1990s, although insufficient computing power hindered initial development, advancements in the backpropagation algorithm and the emergence of the convolutional neural network LeNet enabled neural networks to achieve breakthroughs in image recognition, laying the core algorithmic foundation for modern radiomics and medical image analysis (32, 33).

### 2.5 The era of deep learning (2000s-2020s)

In the 21st century, the explosive growth of computing power and the rise of the Transformer architecture have ushered AI into the era of generative AI and DL (34, 35). During this stage, these technologies have been widely applied in radiotherapy planning, immune response prediction, and the deep integration of multimodal data.

## 3 Applications of AI in cancer radiotherapy

**Figure 2:**
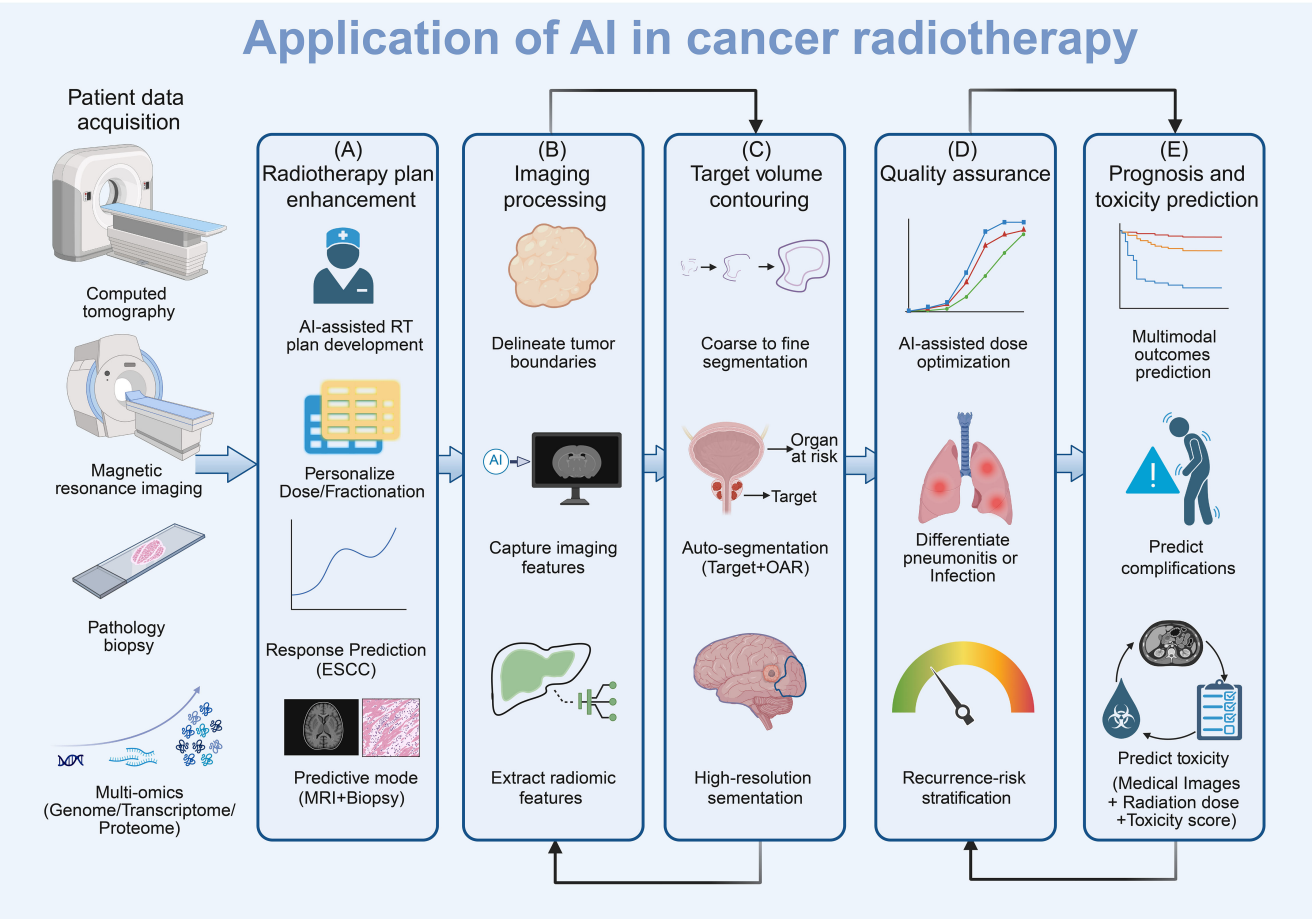
The role of AI in key clinical aspects of cancer radiotherapy, including **(A)** radiotherapy planning enhancement, **(B)** image processing, **(C)** target volume contouring, **(D)** quality assurance, **(E)** prognostic assessment and toxicity prediction.

The seamless integration of AI into radiotherapy is primarily attributable to its highly efficient data processing capabilities and promising clinical application prospects. As a clinical discipline profoundly integrated with computer science, radiotherapy includes a series of complex and highly repetitive workflows, such as treatment planning and optimization, medical image processing, and target volume contouring (36). With the rapid increase in medical imaging and multi-omics data, AI can automatically extract valuable information, identify underlying patterns, and optimize decision-making processes through the radiotherapy workflow (37), which provides the technological foundation for achieving automation and intelligent systems in radiotherapy. These advancements effectively enhance the accurate segmentation of tumor targets and organs-at-risk, as well as accelerate the overall radiotherapy process (38). Additionally, AI significantly enhances the precision and personalization of radiotherapy. By integrating multi-omics data and biomarker analysis, AI can predict treatment outcomes, improve dose distribution, and facilitate the development of patient-specific treatment plans (37, 39). This section details the integration of AI technology across five core stages of radiotherapy, from the initial data acquisition to final prognostic assessment (**Figure 2**), highlighting its comprehensive potential in enhancing the accuracy, automation level, and individualized decision-making. Furthermore, a systematic review of key recent literature on the use of AI—particularly ML and DL—to optimize radiotherapy plans is presented (**Table 1**), covering multiple technical dimensions such as dose prediction, treatment plan optimization, image processing, and individualized dose segmentation strategies.

**Table 1:** Quantitative summary of AI applications in radiotherapy, including dataset/sample size, objective performance metrics, validation level, and limitations.

Role	Cancer	Model	Training input and sample size	Description	Objective performance metrics	Validation/evidence level	Limitation	Reference
Enhancing radiotherapy plan	NSCLC	Γ-LQ and D3QN-based controller	CT scan images of 95 patients who were in the locally advanced stage III of NSCLC and received radiotherapy	Simulating tumor growth and radiotherapy sensitivity using a virtual environment	No AUC/accuracy reported; DRL outperformed current clinical practice in a virtual RT environment	Retrospective/in silico optimization using CT-derived virtual environment; no external clinical validation	Reliance on highly idealized virtual environments	(41)
	ESCC	Six types of CNNs	Pre-treatment CT images of 161 ESCC patients and 70 external validation images	Predicting the situation of pathological complete response after nCRT	External testing: AUC 0.805 (95% CI 0.696-0.913); accuracy 77.1%.	Retrospective two-institution study with external testing cohort (n=70)	Cross-center parameter heterogeneity	(42)
	CRC	ML	The MRI scans and the H&E-stained biopsy sections before treatment	Predicting complete pathological remission	RAPIDS AUC 0.868 training, 0.860/0.872 external validation, 0.812 prospective validation sensitivity 0.888, specificity 0.740	Multicentre retrospective training/validation plus multicentre prospective validation	PPV is relatively low	(36)
Image processing	NSCLC	SDA	A clinical NSCLC dataset consisting of 16 patients and 96 longitudinal CBCT scans	The dynamic changes in tumor shrinkage	Average Dice 0.92 for next-step segmentation; future prediction up to 5 weeks with Dice reduction 0.05; pneumonitis risk reduced up to 35%	Clinical NSCLC dataset (16 patients, 96 longitudinal CBCTs); no external cohort	Extremely limited sample size	(45)
	Small HCC	AI automatic segmentation model	Charged gold nanoparticle-targeted CT images	Automatically assist in identifying and segmenting tumors in CT images	3D U-Net/3D Trans U-Net assessed by accuracy, sensitivity, specificity, AUC, DSC, HD and UoI; exact values not stated in PubMed abstract	Preclinical *in situ* sHCC mouse imaging; 10-fold cross-validation; no clinical validation	Suboptimal accuracy for clinical integration	(46)
	ESCC	ML	MRI images of 70 ESCC patients who underwent CRT curative surgery	Three-dimensional semi-automatic segmentation and extraction of radiomics features from segmented tumors	ADC skewness showed the highest predictive performance (AUC 0.77)	Retrospective single-centre cohort (n=70); stratified double cross-validation	Retrospective, single-center study	(47)
Target volume contouring	/	DL and Lightweight Automatic Cutting Framework RTP-Net	Large-scale CT-related data from 28,581 cases	integrating the CT scanning, contouring, dosimetric planning, and image-guided *in situ* beam delivery in one visit	Average Dice 0.93 +/- 0.11 on 65 OAR tasks; 42/65 tasks >0.90 and 57/65 >0.80; broader experiments report average Dice 0.95	Large-scale public multi-centre data; OAR testing set ~17% (4,833/28,219 images) plus tumor data	Insufficient clinical applicability	(121)
	PRC	DL	Clinical data of 467 patients who received ultra-low-dose radiotherapy for prostate cancer	segmentation of the prostate CTV and the rectum as an OAR	Dataset resource; no predictive AUC/accuracy reported	Open benchmark dataset: 432 prostate patients plus extended 35-patient subset	/	(49)
	Brain tumor	CNN and high-resolution multi-resolution network	The CT or MRI images of the head of patients with brain tumors	Automatically segmenting the anatomical brain barrier for the spread of brain tumor cancer	Average Dice 86.2%; average ASSD 0.499 mm on MICCAI 2020 ABCs challenge data	MICCAI 2020 ABCs challenge data; challenge/testing setting, no prospective clinical validation	Limited organ-specific applicability	(50)
Quality assurance	Cervical cancer	DL and ML	CT images and plans of 671 oART surgeries for 24 cervical cancer patients	Identify measurement deviations that exceed the predetermined standards	ML AUC 0.884, accuracy 0.825; DL_C AUC 0.917, accuracy 0.869; weighted model accuracy 0.855 vs physician consensus 0.795	Model development 588 fractions/21 patients; independent evaluation 83 fractions/3 patients	Small sample size	(52)
	/	Five ML models	The chest CT images of 455 patients	Distinguishing between CIP caused by tumor medication, RP and IP	Test AUCs: RP vs COVID-19 0.92; CIP vs COVID-19 0.68; CIP vs IP 0.71; RP vs IP 0.81; CIP vs RP 0.80; mixed ICI+RT AUC 0.54.	Multicentre cohort (455 patients); mixed ICI+RT test cohort limited	Retrospective design and binary classification model	(53)
	ESCC	DL	A combined radiation imaging map of 302 ESCC patients	Predict local recurrence-free survival rate	Hybrid nomogram C-index 0.82 training, 0.78 internal validation, 0.76 external validation	Training n=201, internal validation n=101, external validation n=95 from two other centres	Small sample size	(54)
Prognostic efficacy and toxicity prediction	Primary lung cancer	DL	CT images and clinical factors of 135 patients with stage T1–2 lung cancer	Determine LRFS, DFS and OS	AUCs: LRFS 0.72, DFS 0.70, OS 0.66	External validation of an existing CT-based model in 135 SABR-treated lung cancer patients	Single-center retrospective design with insufficient follow-up for long-term survival analysis	(56)
	NSCLC	CNN and global average pooling layers	261 CT images of lung cancer patients after processing and trimming	Predicted the SP of lung cancer patients who received radiotherapy combined with immunotherapy	Multimodal model AUC 0.922; radiomics 0.811; clinical 0.711; radiomics+clinical DNN 0.872	Retrospective single-centre cohort (261 patients) with five-fold cross-validation	Single-center retrospective design and lack of biological factors	(57)
	HNSCC	Multimodal DL model	The CT and pathological images of 1087 patients	Predicting prognosis and postoperative radiotherapy response	External C-indices for OS/DFS: 0.717/0.674, 0.713/0.721, 0.682/0.747; TCIA OS C-index 0.636	Multi-centre HNSCC cohorts plus TCIA external cohort	Poor generalizability	(58)
	/	CNN	The dose distribution, CT and abdominal structure scan images of 315 cancer patients	Prediction, localization and interpretation of toxicities after pelvic radiotherapy	CNN-based MIL model predicted toxicity with 80% accuracy	315-patient pelvic radiotherapy dataset; unseen-dataset generalization reported	/	(59)

NR, not reported.

### 3.1 AI in enhancing radiotherapy planning

In recent years, AI has significantly advanced radiotherapy by shifting its foundation from traditional, experience-based clinical practices toward a more individualized, data-driven approach. By analyzing large volumes of medical data, AI enables clinicians to develop personalized radiotherapy plans that better meet the unique biological characteristics of each patient (40). For instance, a recent study showed that a controller based on deep reinforcement learning can formulate personalized treatment plans for patients with non-small cell lung cancer (NSCLC) by analyzing individual CT scan data. This approach overcomes the limitations of one-size-fits-all dosing while also providing a theoretical basis for optimizing daily fractionated treatment (41). Hu et al. reported that DL models trained on CT image datasets exhibit superior performance in assessing treatment responses in patients with esophageal squamous cell carcinoma (ESCC) when compared with models based only on clinical data (42). Additionally, a multicenter observational study confirmed that ML can effectively predict the timing of pathological complete response after radiotherapy in patients with locally advanced rectal cancer (36). However, it is crucial to note that the majority of these applications rely on retrospective, single-center datasets with insufficient sample size, which greatly limits the generalizability of the models compared to robust, large-scale multi-center validations. Overall, these findings indicate that AI-generated insights are increasingly serving as reliable and actionable references in clinical oncology, contributing to the refinement and personalization of radiotherapy plans. Nevertheless, while deep reinforcement learning models show theoretical superiority in dose optimization, they often struggle to significantly outperform traditional physics-based algorithms in prospective clinical trials due to the complexity of real-world anatomical variations. Therefore, their current clinical value lies in serving as an auxiliary decision-support tool rather than an independent planning system.

### 3.2 Image processing

Since its integration into the field of medical imaging, AI has gradually emerged as a key driving force for revolutionizing image processing, by effectively shortening the time required for image processing and enhancing the extraction of key information from the images (43, 44). In the context of adaptive radiotherapy for tumors, a DL model has demonstrated the ability to accurately delineate tumor boundaries and effectively capture complex texture patterns and imaging features, which contributes to reducing the occurrence of radiotherapy-related complications such as radiation pneumonitis (45). A recent preclinical study using a murine model of hepatocellular carcinoma (HCC) has further illustrated that CT contrast agents can achieve accurate and automatic segmentation of HCC lesions in CT images through AI models, thus overcoming the challenges in cancer identification and automatic segmentation in radiotherapy (46). However, the anatomical differences between mice and humans mean that it will take some time before these models can be applied in clinical settings. Furthermore, in patients with advanced esophageal cancer, AI has been successfully applied to perform semi-automatic segmentation of three-dimensional magnetic resonance imaging (MRI) data. Following segmentation, radiomic features are extracted from the delineated cancer regions, enabling researchers to accurately predict the efficacy of radiotherapy (47). Consequently, the images processed by AI are becoming an important reference for clinicians. This trend has enhanced the accuracy of radiotherapy, but current models often fails to meet the performance requirements across different populations, hospitals, and imaging modalities.

### 3.3 Target volume contouring

Accurate delineation of the target volume is a crucial step in the delivery of effective and safe RT for patients with cancer (48). Traditional manual contouring is time-consuming and subject to inter-observer variability, which can compromise both therapeutic precision and patient safety. Notably, recent advances in AI have significantly improved the efficiency and accuracy of target volume contouring. Shi et al. proposed a DL-based framework that can facilitate the initial segmentation of systemic tumors, enabling a transition from coarse to fine segmentation. Moreover, this framework demonstrates adaptability across tumors of varying sizes and boundary characteristics, thereby assisting clinicians in rapidly identifying tumor regions (48). Further research has applied AI to automate the segmentation of both target volumes and critical organs at risk in prostate cancer (49). Additionally, a study has introduced a two-stage AI-driven segmentation strategy that preserves high resolution while precisely segmenting the margins of cancer cell spread in the brain. This method is particularly effective for delineating thin or low-contrast structures that are difficult to visualize manually (50). In the current era of DL, automated tumor localization has become increasingly feasible, thereby reducing the reliance on manual intervention. This shift promotes the development of radiotherapy toward personalized, precision-based treatment strategies.

### 3.4 Quality assurance

Radiotherapy is a highly complex process involving multiple steps, each of which requires rigorous quality assurance to ensure treatment accuracy and patient safety. ML has made significant strides in the field of quality assurance across various phases of radiotherapy in recent years (51). A recent study on cervical cancer patients in 2025 showed that AI, by identifying key features such as the uterus and cervix from medical images, achieved more precise adaptive dose optimization compared to decisions made by human physicians (52). Moreover, during the course of radiotherapy, ML has also shown utility in monitoring treatment-related complications. ML is capable of effectively distinguishing between the highly similar conditions of radiation pneumonitis and infectious inflammation during chest radiotherapy, thereby assisting clinicians in real-time assessment of the treatment safety and facilitating timely interventions (53). Following treatment completion, AI contributes to long-term patient management and recurrence prevention. Gong and colleagues developed a hybrid radiomics prediction model based on DL for patients with ESCC. Specifically, this model can classify patients into high- and low-risk groups for local recurrence, thereby enabling early intervention and ensuring the long-term safety of radiotherapy (54). In the future, AI may incorporate quality control into the entire process of radiotherapy, thereby reducing human error, enhancing treatment consistency, and ultimately improving therapeutic outcomes.

### 3.5 Prognostic assessment and toxicity prediction

In the personalized and precise implementation of radiotherapy, accurate prediction of treatment outcomes and potential toxicity is essential for formulating treatment plans and balancing efficacy and safety (55). AI plays a central role in this process by constructing high-dimensional predictive models that integrate diverse clinical data. For instance, a DL-based model trained on CT images has demonstrated excellent performance in predicting post-treatment outcomes for patients with lung cancer, showing both high accuracy and robust external validity across different clinical settings (56). Moreover, in the treatment of NSCLC, a multimodal DL model can predict the occurrence of complications in patients receiving combined radiotherapy and immunotherapy, which not only reduces radiation-induced lung injury but also contributes to prolonged long-term survival outcomes (57). A recent study involving over 1,000 patients with head and neck squamous cell carcinoma further revealed that a multimodal DL model can effectively predict the overall survival and disease-free survival by analyzing features from the tumor microenvironment (TME) and metabolites (58). Regarding toxicity prediction, Elhaminia et al. proposed a convolutional neural network that can precisely predict abdominal and iliac bone toxicity in patients receiving pelvic radiotherapy by analyzing medical images, radiation doses, and toxicity scores (59). Overall, the true value of AI in this field lies in its ability to synthesize complex, high-dimensional datasets. However, while retrospective studies show that AI can accurately predict progression-free survival (PFS) and overall survival (OS), prospective trials demonstrating that AI-guided interventions directly improve these hard clinical outcomes compared to standard care remain scarce. AI assists clinicians in identifying the optimal balance between therapeutic efficacy and toxicity, providing a theoretical foundation for the future development of intelligent adaptive radiotherapy strategies (60).

## 4 Applications of AI in cancer immunotherapy

While AI has fundamentally reshaped the precision of local tumor control through optimized radiotherapy planning, the clinical reality of oncology demands a transition from local intervention to systemic management. The radiomic signatures and target-volume dynamics captured by AI during radiotherapy provide more than just geometric guidance; they offer profound insights into the tumor’s biological behavior and phenotype. This granular understanding of tumor heterogeneity serves as the bridge to the next critical pillar: leveraging AI to predict systemic treatment responses, particularly in the realm of personalized immunotherapy, where the digital decoding of the TME becomes the determinant of therapeutic success.

**Figure 3:**
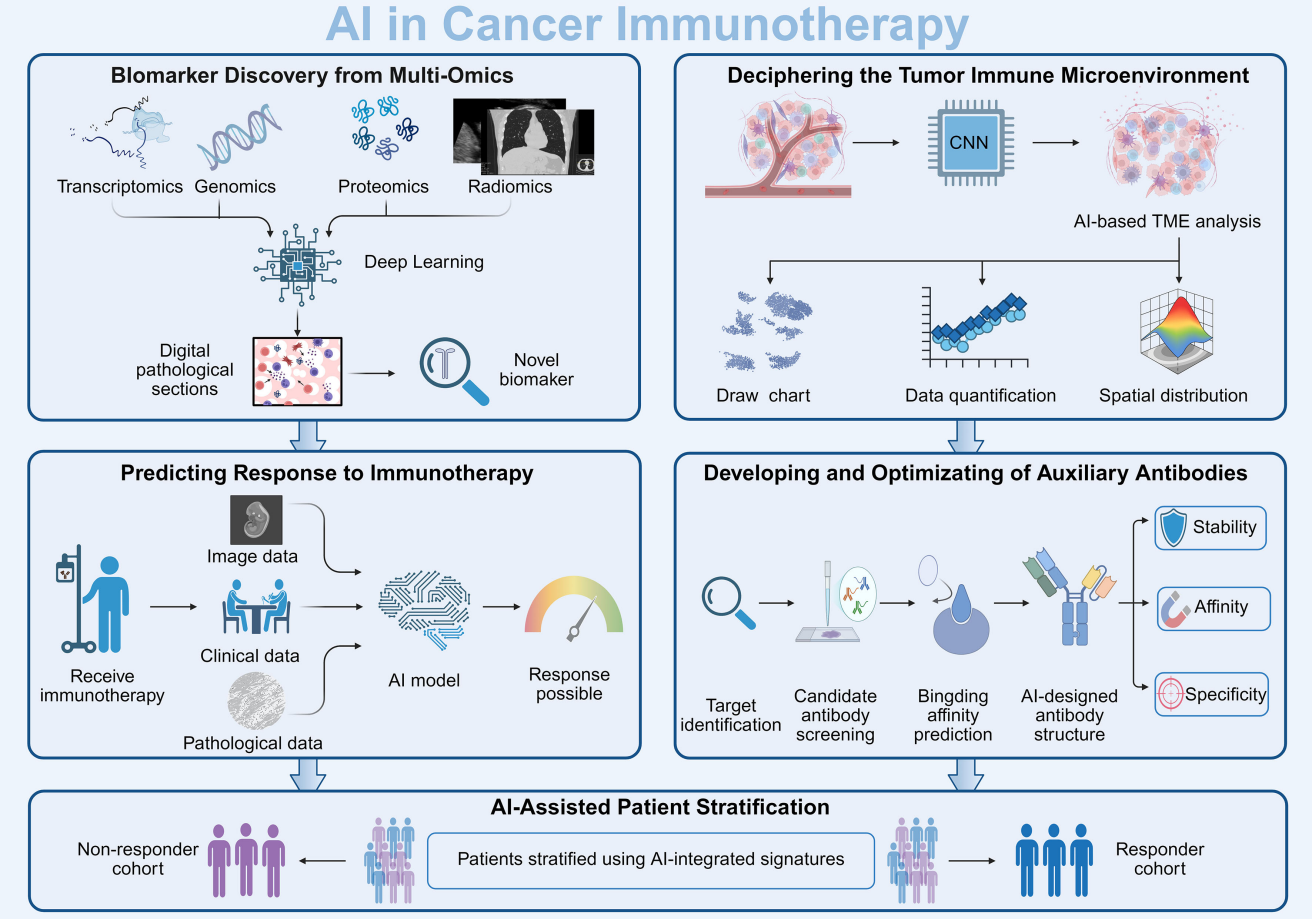
The application of AI in the tumor immunotherapy process across molecular mechanism, the tumor microenvironment, and patient perspectives. CNN, Convolutional Neural Network.

The integration of AI and immunotherapy in oncology is driven by the inherent complexity of immunotherapeutic regimens and the distinct capabilities of AI in processing high-dimensional data. Recent advancements in immunotherapy, particularly the application of ICIs such as programmed cell death 1 (PD-1) or programmed death ligand-1 (PD-L1) inhibitors, have achieved substantial improvements in patient outcomes (61, 62). However, the therapeutic efficacy of these treatments exhibits considerable heterogeneity, with only a subset of patients demonstrating a positive response to ICIs (63). The unpredictability of immunotherapy responses has thus become a limiting factor in clinical practice. This necessitates the development of precise tools for patient stratification and the prediction of treatment efficacy. The potential of AI technologies, with their advanced data processing and pattern recognition capabilities, offers a promising solution to these challenges (24). Furthermore, AI has demonstrated its capacity to analyze massive amounts of biological data, leading to the identification of novel biomarkers and therapeutic targets associated with immunotherapeutic efficacy (15). Therefore, the application of AI in immunotherapy has provided new directions for the oncological management, driving a critical transition from broad phenotypic observations to the precise decoding of granular, cell-specific immunomodulatory mechanisms. This section systematically details how AI plays a role in key aspects such as biomarker discovery, analysis of the tumor immune microenvironment, efficacy prediction, and the development of auxiliary antibodies (**Figure 3**), aiming to demonstrate the comprehensive integration capability of AI across molecular, cellular, and clinical levels. Furthermore, a systematic summary of key recent research achievements regarding AI technology in the field of cancer immunotherapy is provided (**Table 2**), with a focus on TME applications, immune response prediction, and antibody screening.

**Table 2:** Application of AI in immunotherapy.

Role	Cancer	Model	Training input	Description	Limitation	Reference
Deciphering the tumor immune microenvironment	SCLC	ML	100,544 pathological images of 8 patients	Analyze the TIME prediction for 1-year PFS	Absence of independent external validation	(75)
Predicting the response to immunotherapy	ESCC	Multimodal DL model	Pathological and CT images as well as clinical factors of 295 patients who received immunotherapy	Predicting PD-L1 levels, immune treatment response and overall survival rate	Binary classification constraints	(77)
	ESCC	ViT-RNN	The WSI images of 324 H&E-stained histological specimens from 163 patients	Predicting the clinical benefits of PD-1 inhibitors in patients with ESCC	Lack of external cohort validation	(78)
	GC	Seven Types of ML	The radiopathological characteristics of 298 GC patients	Predict the therapeutic response and stratify the prognostic risk of GC	Retrospective nature and selection bias	(63)
	GC	DL	Clinical and imaging data of 168 patients who received immunotherapy	By predicting the response to immunotherapy, we can then predict the biomarkers	Dimensionality constraints of 2D input	(79)
Development and Optimization of Auxiliary Antibodies	GBM	Meta-analysis and ML	Weighted gene co-expression analysis and clinical immunotherapy dataset	Identify candidate genes related to the initiation, progression, prognosis and immune treatment response of GBM	Limited experimental validation	(81)
	Diffuse glioma	Eighteen ML algorithms	A meta-analysis of the prognosis of 2968 patients with diffuse glioma	Predict the survival outcomes of various cancers and stratify patients accordingly	/	(82)
Assist in patient classification	GBM	ML	The genetic maps of 868 patients	Classify the patients into two dryness subtypes	Small institutional validation cohort	(88)
	HCC	ML	The cancer genome and RNA-seq datasets of 596 patients	Divide HCC patients into two dryness subtypes	Limited neoadjuvant immunotherapy cohort	(89)
	/	CNN-LSTM model	Data on RNA expression and DNA methylation of 32 types of tumor patients	Predict the type of tumor and classify it	Sensitivity to high-noise data	(91)
	Low-grade gliomas	10 ML Algorithms	Sequencing data of purified immune cells, LGG cell lines and large pieces of LGG tissue	Establish the TIIClnc signature and select patients with better therapeutic effects	Single-center retrospective design and unclear molecular mechanisms	(93)

### 4.1 Biomarker discovery based on multi-omics data

The rapid development of AI in the field of immunotherapy is profoundly changing the traditional biomarker analysis paradigm and promoting the discovery of new composite biomarkers based on the integration of multi-omics data. At present, PD-L1 expression levels and tumor mutational burden are the most widely used biomarkers for predicting the efficacy of ICIs in clinical practice (64, 65). However, relying on a single biomarker presents limitations such as insufficient sensitivity and specificity, strong spatial heterogeneity, and significant dynamic changes, which limit its clinical guidance value (66–68). To address these issues, AI technology efficiently analyzes massive genomic, transcriptomic, and imaging data through DL models, significantly optimizing the analytical accuracy and repeatability of existing biomarkers. In the field of PD-L1 analysis, AI can overcome substantial inter-observer variability through the automatic recognition and quantitative assessment of digital pathological sections. This approach achieves high-throughput calculation of the proportion of PD-L1 positive cells across the entire field of view, thereby improving the consistency of detection (69, 70). Despite these technical advantages, robust evidence showing that these AI-derived multimodal biomarkers definitively outperform current clinical gold standards—such as standard PD-L1 immunohistochemistry or routine tumor mutational burden profiling—in directing clinical decisions is still lacking.

### 4.2 The application of AI in deciphering the tumor immune microenvironment

The integration of AI in the analysis of the tumor immune microenvironment has emerged as a significant approach in cancer research. The tumor immune microenvironment, a critical component of solid tumor, serves as a crucial determinant in influencing tumor progression and response to therapy (71, 72). Traditional biological methods face significant challenges in capturing the complexity and heterogeneity inherent in the TME due to their limitations in scalability and resolution. AI offers a powerful alternative by constructing data-driven models that efficiently interpret large-scale biological datasets. Ye et al. proposed a convolutional neural network that can extract features from transcriptomic data, providing a more comprehensive landscape of the immune microenvironment (73). Tumor-infiltrating lymphocytes (TILs), which are central to anti-tumor immunity, have traditionally been assessed through histological staining followed by manual cell counting. With the rapid development of AI in recent years, AI-based techniques can now achieve automatic quantification of TILs, identification of subgroups, and analysis of spatial distribution patterns, all of which support the high-resolution mapping of immune cell interaction within the TME at the cellular level (74). This analysis of the spatial architecture helps researchers understand how immune cells are physically isolated by the tumor stroma, thereby revealing the mechanism of physical immune escape. A recent prospective study in patients with small cell lung cancer (SCLC) exemplified this progress by showing that a ML model integrating multimodal data achieved higher accuracy than traditional manual counting assessment This improved performance enables a deeper understanding of TME dynamics and supports the development of more effective immunotherapy strategies (75). Beyond simple cell counting, AI-driven multi-omics integration provides crucial mechanistic depth by mapping complex spatial interactions, such as macrophage chemokine networks, and precisely quantifying functional states like adaptive CD8+ T cell exhaustion, which are essential for identifying novel therapeutic vulnerabilities. In particular, the core advantage of AI in analyzing the TME lies in its ability to handle multi-dimensional and dynamic biological data; this significantly enhances our understanding of TME complexity and is expected to lay the foundation for the realization of individualized immunotherapy strategies in the future.

### 4.3 The application of AI in predicting the response to immunotherapy

In the field of oncology, the use of AI to predict patients’ responses to immunotherapy has become a key focus of research in personalized treatment strategies (76). A recent study involving ESCC introduced a novel DL model incorporating multimodal data—including histopathological features, imaging features, and clinical information—that has demonstrated promising performance in predicting PD-L1 expression levels and immunotherapy responses. This model provides a low-cost, non-invasive immunotherapy prediction tool for medical institutions (77). Furthermore, recent studies have shown that solely relying on histological image analysis can improve the prognosis classification of patients with ESCC. This technique can capture subtle expression levels of PD-L1 that are difficult to detect with the naked eye, thereby predicting the efficacy of PD-L1 inhibitors (78). Given the difficulty in identifying reliable biomarkers for predicting immunotherapy responses, researchers developed an imaging-based model specifically for gastric cancer. This model, by analyzing the immune-regulating pathways and the infiltration of memory B cells, can accurately distinguish patients who are likely to have a favorable response to immunotherapy (63, 79). Nevertheless, these prediction models are predominantly derived from retrospective cohorts; therefore, their evidence weight must be interpreted cautiously until they undergo rigorous prospective validation to confirm their reliability. The precise prediction of immunotherapy responses by AI effectively avoids the toxic side effects and economic burden caused by ineffective treatments and promotes the advancement of precision immunotherapy. However, a critical evaluation of these models reveals that their predictive accuracy currently varies significantly across different cancer types and imaging modalities. When compared with established clinical biomarkers like PD-L1 or TMB, AI models should currently be viewed as complementary tools rather than complete replacements, pending multi-center prospective validation to prove their absolute clinical superiority.

### 4.4 Development and optimization of auxiliary antibodies

AI is reshaping the discovery and optimization of immunotherapeutic antibodies, utilizing its powerful computing capabilities to support the entire process of the antibody development pipeline, from target identification to preclinical evaluation (80). In a recent study, Song et al. applied ML to analyze multi-center transcriptomic profiles and integrated AI-driven drug screening methods, successfully identifying novel antibody candidates (81). Similarly, diffuse glioma, a common and highly aggressive form of brain cancer, remains difficult to treat with conventional therapies. Researchers established a ML framework to screen siRNAs and discovered the crucial role of the TRAF3IP3 target in regulating PD-L1 expression, which provides a molecular biological basis for understanding the immune escape and drug resistance mechanisms of gliomas (82). Further evidence demonstrates that AI models can efficiently predict the interaction between antibodies and targets, calculate antibody properties, and guide the selection of optimal candidates from large the antibody libraries (83). These capabilities significantly shorten the time-consuming antibody development cycle and accelerate the design process, thereby facilitating faster progression toward next-generation therapeutic antibodies. Importantly, AI not only predicts binding affinities but also increasingly models the downstream functional impact of these antibodies on immune cell activation, ensuring that candidates can effectively reprogram the immunosuppressive microenvironment. Additionally, advances in protein structure prediction powered by AI have shown strong practical application potential in therapeutic antibody design. AlphaFold can accurately predict the three-dimensional structures of antibodies and their targets, simplifying the experimental process and enabling the rational design of complementarity-determining regions (84). With the rapid progress of AI algorithms and the growing availability of antibody libraries, the integration of AI into antibody design is expected to act as a key driving force for future antibody development and optimization.

### 4.5 AI-assisted patient stratification

Accurate tumor classification and subsequent patient stratification are essential for identifying individuals who are most likely to benefit from specific immunotherapeutic interventions. AI is playing an increasingly important role in this process by identifying biologically meaningful markers (85–87). Wang et al. applied ML to analyze stem cell-related characteristics and developed a method for distinguishing patient subgroups based on glioma membrane profiles. This method successfully identified a subset of patients with an enhanced potential response to immunotherapy (88). Similarly, another study applied four ML algorithms to distinguish different subtypes of HCC stem cells and constructed a gene-based classifier to identify unique immune phenotypes, thus revealing how tumor stem cells achieve immune evasion by remodeling the microenvironment (89, 90). In addition to stem cell-based classification, the AI-driven analysis of RNA expression profiles has become a widely adopted strategy for tumor characterization (91, 92). A related study involved the utilization of ten ML algorithms and sixteen tumor-infiltrating immune cell-associated long non-coding RNAs (TIICIncRNAs) to construct a predictive TIIClncRNA signature for low-grade glioma. This signature reveals the potential role of non-coding RNA in regulating the biology of immune checkpoints and lymphocyte infiltration, providing molecular evidence for identifying patients who will benefit from immunotherapy (93). Thus, these AI-driven integrated analyses provide clinicians with reliable tools for initial patient stratification.

### 4.6 AI-driven integration of radiotherapy and immunotherapy

The synergistic combination of radiotherapy and immunotherapy represents a critical frontier in modern oncology. Radiotherapy can function as an *in situ* vaccine, reshaping the TME to reverse adaptive CD8+ T cell exhaustion and potentiate the efficacy of ICIs (94). However, predicting clinical response remains challenging due to pronounced immune heterogeneity. AI addresses this bottleneck by integrating multi-omics and radiomics data to decode post-radiotherapy TME dynamics. For instance, AI-driven models in HCC can capture subtle variations in immune infiltration, thereby accurately predicting survival benefits and stratifying patients for immunoradiotherapy (95). Furthermore, AI enables the differential diagnosis of overlapping clinical complications, such as distinguishing radiation pneumonitis from immune-related pneumonitis. Consequently, AI serves as a pivotal tool to optimize patient selection and maximize the synergistic therapeutic efficacy of combined radioimmunotherapy.

A more systematic view of AI-enabled radioimmunotherapy can be organized into three clinical tasks. First, pretreatment models can identify patients most likely to benefit from RT-intensified immunotherapy. Second, toxicity-focused models in lung cancer and NSCLC can combine CT, dosimetric, clinical, and radiomics features to distinguish radiation pneumonitis, immune-related pneumonitis, and infectious inflammation, thereby supporting safer combined regimens. Third, multimodal response-prediction frameworks developed in ESCC and GC immunotherapy cohorts provide a transferable structure for future radioimmunotherapy trials, although direct prospective validation remains limited.

Across cancer subtypes, the common AI strategy is to fuse radiomics, pathomics, multi-omics, dosimetry, and clinical variables rather than relying on a single biomarker. This integrated approach may improve patient selection, schedule optimization, immune-toxicity monitoring, and early identification of non-responders. Nevertheless, prospective trials should report sample size, AUC, accuracy, sensitivity, specificity, calibration, and external validation to establish whether AI improves outcomes beyond standard clinical decision-making.

## 5 Applications of AI in drug discovery and repurposing

However, the AI-driven ability to predict chemotherapy resistance or suboptimal immune responses exposes a persistent clinical bottleneck: the scarcity of effective therapeutic alternatives for refractory patients. This predictive ‘dead-end’ necessitates a strategic shift from merely utilizing existing drugs to actively discovering new ones. By integrating the multimodal data used for treatment response prediction, AI can further extend its utility into the upstream stages of the pharmaceutical pipeline. The following section explores how AI transforms this challenge into an opportunity by accelerating the discovery of novel compounds and the repurposing of existing drugs, thereby creating a continuous loop between clinical bedside observations and laboratory-based drug development.

**Figure 4:**
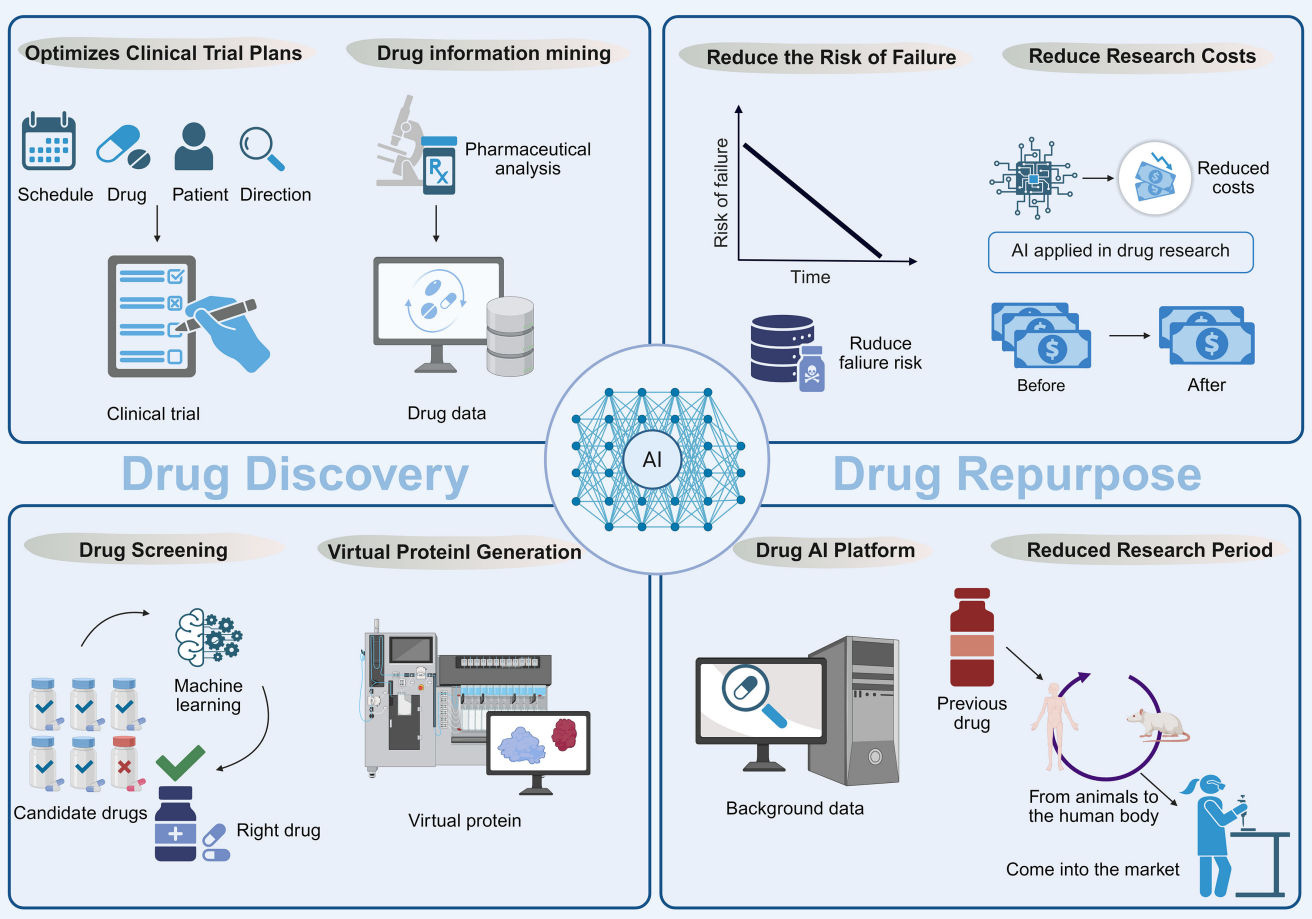
The role of AI in drug discovery and repurposing, encompassing drug data collection, drug design, and the evaluation of clinical efficacy.

AI is revolutionizing drug discovery and repurposing by rapidly analyzing vast biological datasets to predict drug-target interactions, identify novel compounds, and uncover new therapeutic uses for existing medicines, thereby significantly accelerating the development timeline. This section illustrates how AI can enhance the efficiency of the entire drug development lifecycle, from candidate molecule screening, and virtual protein generation to clinical trial optimization (**Figure 4**), aiming to emphasize the strategic value of AI in reducing development risks, shortening development cycles, and minimizing economic costs. Furthermore, representative research on AI technology in the search for new anti-tumor molecules and the exploration of new indications for existing drugs is systematically summarized (**Table 3**), highlighting the practical contributions of ML and DL models in enhancing the success rate of drug development, reducing trial costs, and shortening the translational cycle.

**Table 3:** Application of AI in drug discovery and repurposing.

Cancer	Model	Training input	Description	Limitation	Reference
/	DL	Information about STK33-related drugs	Antiviral drug Z29077885	Preliminary stage of drug development	(101)
NSCLC	Transfer learning	Data related to tyrosine kinase inhibitors	Drug-like ligands targeting the L858R/T790M/C797S mutations of EGFR	Undetermined covalent binding affinity and selectivity constraints within kinase families	(103)
/	The framework for comprehensible graphical prediction of drug reactions (XGDP)	The potential characteristics of drugs represented by molecular graphs	Predicting drug response	GPU resource constraints and suboptimal hyperparameter optimization	(104)
Lung cancer	GAN and DL	Genes associated with adverse clinical outcomes in lung cancer patients	A number of small molecule drug formulations	Potential discrepancy between prediction and reality	(105)
Breast cancer	Seven DL prediction models	Three different differentiated subgroups of Tregs	The synergistic effect between drugs	Reliance on computational transcriptomics	(108)
/	Intelligent learning engine	Drug-related molecular/protein dataset	Retrieval, analysis and comparison of molecular data	Domain-specific generalizability constraints	(111)
/	FP-GNN DL	485,900 kinds of compounds involve 3,919,974 biological activity records	The server for completing tasks related to the discovery of anti-cancer drugs	/	(109)
ccRCC	CNN and DL	The scRNA-seq data of six patients	Five specific compounds for CC RCC that can be used in drug development	Extremely small cohort size:	(113)
/	DeepDRK	Over 20,000 pairs of cancer cell lines - anti-cancer drug information	Analyzing drug reactions and predicting the clinical responses of cancer patients	/	(118)
HCC	MDeePred DL	A dataset of 380 drug-target interaction data	Six compounds that can bind to HCC targets	/	(119)
HCC	Seven ML algorithms	Biological markers genes related to the staged progression of HCC associated with NAFLD	81 candidate drugs that are effective for biomarkers	Transcriptomic-only profiling bias:	(120)

### 5.1 Drug discovery

The development of drugs is regarded as a pivotal process for transforming scientific research into clinical applications. However, developing new drugs is a highly complex and time-consuming endeavor (96). The integration of AI, particularly DL, has emerged as a transformative force in accelerating key stages of drug discovery (97–99). By analyzing large-scale datasets via advanced computational models, AI enhances target identification, compound screening, and clinical trial design, thereby significantly improving efficiency, reducing failure rates, and controlling costs (100, 101). In terms of target discovery, DL models have demonstrated the ability to rapidly identify novel disease-associated molecular targets and biological pathways through virtual screening techniques, thereby guiding new drug development (102–104). In compound screening, AI models, particularly graph neural networks (GNNs), integrating multi-omics data has shown strong predictive power in estimating the binding affinity between small molecules and their target proteins (105). For instance, Duo et al. found that AI-driven analysis enables the efficient identification of small-molecule candidates with anti-cancer potential (106). Similarly, a recent study in breast cancer has revealed that the combination of AI and computer-aided drug design (CADD) overcomes the shortcomings of high resource requirements and low time efficiency associated with traditional CADD, thereby improving its overall efficiency and scope (107). Further evidence supports the application of AI in generating predictive prognostic models by analyzing interactions between regulatory T cells (Tregs) and the TME. DL-based analysis of these data and prognostic features has revealed synergistic effects among therapeutic agents, offering new insights into overcoming immune resistance mechanisms in cancer therapy (108). A notable example is the construction of 832 DL models trained on a dataset comprising 485,900 compounds. Based on a fingerprint-based graph neural network (FP-GNN), researchers predicted the inhibitory activity of these compounds against tumors and developed the DeepCancerMap web server, which support researchers in identifying promising anti-cancer compounds through data-driven screening (109). Beyond small molecules, DL is able to predict the three-dimensional structure of proteins with extremely high accuracy and simulate their dynamic changes and interactions among proteins, thereby providing a crucial structural basis for designing highly specific and active protein compounds (110, 111). AlphaFold, an AI model developed for predicting protein structures, has achieved unprecedented accuracy in determining three-dimensional protein conformations from amino acid sequences alone. This breakthrough was recognized with the 2024 Nobel Prize in Chemistry and has profoundly impacted drug discovery by providing precise structural templates for rational drug design (112). Wang et al. employed single-cell analysis combined with virtual screening to identify potential therapeutic compounds targeting clear cell renal cell carcinoma (ccRCC)-specific protein structures. Subsequently, they screened anti-ccRCC compounds from a bioactive compound library using CNN and ML, and ultimately identified five ccRCC-specific compounds (113). Moreover, AI plays an increasingly vital role in optimizing clinical trial processes. By analyzing real-world evidence, AI facilitates more precise patient selection based on biomarkers and clinical profiles. It enhances patient recruitment efficiency, improves stratification strategies, accelerates data analysis during trials, and enables the earlier detection of treatment efficacy or adverse events (114). As technology continues to advance, AI is gradually evolving from an auxiliary tool to the primary driving force in drug research, leading the pharmaceutical industry towards a more intelligent, precise, and efficient new era.

In the specific realm of cancer immunotherapy, AI is reconciling the critical gap between target identification and clinical translation, particularly for ICIs and therapeutic antibodies. While traditional monoclonal antibody development remains resource-intensive, DL models can now predict antigen-antibody binding affinities and optimize complementarity-determining regions to engineer highly specific bispecific antibodies or nanobodies. Beyond macromolecular therapeutics, AI-driven virtual screening and high-precision molecular docking are accelerating the discovery of small-molecule immunomodulators. For instance, computational platforms can identify novel compounds that modulate the innate macrophage chemokine network or reverse adaptive CD8+ T cell exhaustion. By achieving precise structural alignment, such as targeting specific amino acid residues in immune target pockets, AI ensures superior binding accuracy for these novel candidates. Furthermore, AI-guided pharmacological evaluations can forecast complex drug interactions; for example, they can accurately simulating whether a specific antagonist will partially or entirely abrogate the protective efficacy of an immunotherapeutic intervention, thereby streamlining the optimization of novel drug regimens. Yet, the transition from AI-predicted binding affinities to actual *in vivo* efficacy remains a significant hurdle. Current AI approaches often fail to fully account for complex pharmacokinetic and pharmacodynamic interactions in the human body, meaning that while AI excels at rapid screening, its true value in reducing late-stage clinical trial failure rates still requires robust real-world validation.

### 5.2 Drug repurposing

Drug repurposing refers to the process of identifying new therapeutic indications for drugs that have already been approved for other conditions or have undergone clinical investigations. Compared to the traditional *de novo* drug development path, it can significantly reduce development time and costs while improving the likelihood of clinical success by leveraging pre-existing data on the safety and pharmacokinetics of known drugs in the human body. The rapid development of AI has provided new solutions for systematically and massively identifying opportunities for drug repurposing through the integration of big data analysis (115–118). A notable example is the AIDDISON platform, which supports systematic screening for drug repositioning opportunities across various conditions. In an HCC study (119), researchers used a ML model called MDeePred to analyze interactions between drugs and their molecular targets. The model successfully identified 6 candidate drugs that closely matched approved late-stage HCC treatments in terms of efficacy profiles and target specificity. Additionally, another study used an integrated feature selection framework including seven ML algorithms to analyze transcriptome data, precisely identifying potential biomarker genes related to the progressive stages of non-alcoholic fatty liver disease (NAFLD)-related HCC. These findings provided clear targets for drug repurposing, and efficiently filtered effective candidate drugs for these biomarker genes, thereby enhancing the targeting and efficiency of drug repurposing (120). In summary, AI is transforming drug repurposing from an accidental, experience-driven exploration into a systematic, data-driven discovery. Its promising application lies in identifying the immune-enhancing properties of non-immune drugs, thereby providing new combination therapy options for ICIs.

Furthermore, the integration of AI in drug repurposing holds profound implications for advancing cancer immunotherapy. By analyzing transcriptomic data and immune cell infiltration profiles, ML models can systematically screen for approved non-oncology drugs with unrecognized immunomodulatory properties. Repurposing these existing agents to reprogram tumor-associated macrophages or modulate the TME provides a rapid, cost-effective pipeline to overcome ICI resistance and achieve synergistic effects with current immunotherapies.

## 6 Conclusion and future perspective

The deep integration of AI into the medical field has transcended its initial role as a mere auxiliary technology. It is now gradually becoming a driving force shaping the future of healthcare, especially in the field of tumor immunology. This review systematically provides a comprehensive overview of AI’s multifaceted contributions throughout the entire chain of tumor treatment and research. AI plays a critical role in precisely delineating target areas, optimizing radiotherapy plans, and analyzing the complex TME to predict responses to immunotherapy. Furthermore, it enables detailed patient stratification and prognosis prediction through the integration of multi-omics data, thereby supporting personalized treatment strategies. In drug development, AI accelerates both novel drug discovery and the repurposing of existing pharmaceuticals by efficiently analyzing large-scale chemical and biological datasets. These applications collectively underscore a core paradigm shift: medicine is transitioning from a traditional, experience-driven model to a new era founded on data-driven and algorithmic insights.

However, substantial challenges remain hindering the widespread clinical adoption of AI. A significant limitation across the current literature is the lack of evidence stratification, as many studies present preliminary algorithmic successes without high-level clinical validation. Currently, the training datasets for most models are primarily derived from single-center cohorts, lacking the integration of large-scale, multi-center data, which inherently restricts model generalizability. Additionally, the absence of external validation poses challenges to model reproducibility in real-world applications. Finally, the dearth of prospective clinical trials, coupled with an unclear division of legal and ethical responsibilities, creates significant barriers to clinical translation. Consequently, very few of these AI-based approaches have been formally incorporated into international oncology guidelines or routine clinical workflows, as regulatory bodies necessitate rigorous, randomized evidence of improved patient outcomes. Data quality and standardization still lack rigorous protocols, which requires the establishment of robust ethical guidelines and regulatory frameworks that ensure transparency, fairness, and accountability in AI applications.

These future directions should also be interpreted with realistic translational boundaries. Spatial transcriptomics and single-cell platforms remain expensive and require specialized tissue processing, sequencing, imaging, and bioinformatics infrastructure. Oncology digital twins depend on continuous longitudinal data capture, interoperable electronic health records, stable imaging pipelines, and secure multi-institutional data governance. Generative AI-based drug development must overcome wet-lab validation, pharmacokinetic uncertainty, intellectual-property ownership, regulatory review, and commercial scalability before routine clinical deployment.

Moving forward, the true potential of AI lies in its deep integration with cutting-edge interdisciplinary technologies. First, the convergence of AI with high-resolution spatial transcriptomics and single-cell RNA sequencing (scRNA-seq) will enable the dynamic 3D reconstruction of the tumor immune microenvironment. This interdisciplinary approach allows researchers to decode complex cell-to-cell interactions and metabolic-immune crosstalk at an unprecedented single-cell resolution, moving beyond bulk tissue analysis to identify rare therapy-resistant cellular subpopulations.

Second, the integration of continuous multi-omics, longitudinal imaging, and clinical data is driving the conceptualization of “Oncology Digital Twins.” These dynamic computational replicas of individual patients will allow clinicians to simulate disease progression and predict the efficacy of various radioimmunotherapy combinations in silico before administering them *in vivo*.

Finally, as generative AI matures, its application in pharmacology will transition from predicting known protein structures to the *de novo* design of multi-specific immunomodulators. By targeting precise molecular vulnerabilities and key metabolites regulating the TME, these AI-generated compounds hold the promise of reversing immune exhaustion and overcoming current therapeutic bottlenecks. Ultimately, AI is not aiming to replace clinicians, but rather to serve as an indispensable interdisciplinary platform that translates vast biological complexity into precise, actionable therapeutic strategies.
